# Plant-based dietary indices and mental health: a cross-sectional study of a middle- to older-aged population

**DOI:** 10.1007/s00394-026-04000-z

**Published:** 2026-05-26

**Authors:** Seán R. Millar, Ivan J. Perry, Catherine M. Phillips

**Affiliations:** 1 School of Public Health, University College Cork, Cork, Ireland; 2 School of Public Health, Physiotherapy and Sports Science, University College Dublin, Dublin 4, Ireland

**Keywords:** Plant-based diets, Mental health, Depression, Anxiety, Well-being

## Abstract

**Purpose:**

Plant-based diets (PBDs) have been linked with favourable cardiometabolic health outcomes. However, there has been limited investigation of PBD indices (PDIs) and mental health outcomes. Addressing this gap, we examined an overall PDI (oPDI), healthy PDI (hPDI) and unhealthy PDI (uPDI) associations with depressive symptoms, anxiety and well-being.

**Methods:**

This cross-sectional study includes 1,949 middle- to older-aged men and women from the Mitchelstown Cohort. PDIs were calculated from validated food frequency questionnaires. Depressive symptoms, anxiety and well-being were assessed using the 20-item Center for Epidemiologic Studies Depression (CES-D) Scale, the anxiety sub-scale of the Hospital Anxiety and Depression Scale and the World Health Organization-Five Well-Being Index. Regression analyses investigated PDI relationships with mental health scores, adjusted for potential confounders.

**Results:**

In age and sex-adjusted models, the oPDI and hPDI were negatively associated with depressive symptoms (β = −0.095, 95% CI: −0.154, −0.036; *p* = 0.002 and β = −0.086, 95% CI: −0.136, −0.035; *p* = 0.001, for oPDI and hPDI scores respectively), and positively associated with greater well-being (β = 0.045, 95% CI: 0.006, 0.084; *p* = 0.025 and β = 0.037, 95% CI: 0.003, 0.071; *p* = 0.032, for oPDI and hPDI scores respectively). Additionally, the oPDI was inversely associated with anxiety (β = −0.027, 95% CI: −0.053, −0.002; *p* = 0.038). Higher oPDI and hPDI score associations with fewer depressive symptoms, and oPDI score associations with anxiety, persisted upon full adjustment, with PDI quartiles demonstrating significant dose-response relationships with CES-D scores (*p* trend < 0.05).

**Conclusions:**

Greater adherence to an overall PBD, and more healthful PBD, is associated with fewer depressive symptoms and greater well-being among middle- to older-aged adults. Future longitudinal studies which explore causal relationships between PDIs and psychological outcomes are warranted.

**Supplementary Information:**

The online version contains supplementary material available at 10.1007/s00394-026-04000-z.

## Introduction

Mental health disorders are a growing public health concern with an estimated 300 million people globally living with depression, while anxiety disorders impact approximately 264 million people worldwide [1]. These disorders significantly contribute to the global burden of disease through years lived with disability [2], impairing quality of life, with a concomitant economic burden arising from associated healthcare costs and lost productivity [3].

The interplay between diet and mental health has garnered increasing attention in recent years, reflecting a broader understanding of how lifestyle factors contribute to overall well-being [4]. In recent years the focus of nutritional research has shifted from studying individual foods and nutrients to examining dietary patterns and scores that indicate habitual eating habits. These reflect the fact that foods are eaten in combination, thus removing the limitation that single nutrients may not reveal the overall quality of diet as a whole and that disease processes are often influenced by the overall composition of a diet, not just single nutrients [5].

There is a growing body of evidence suggesting that healthy diets, particularly greater adherence to the Mediterranean diet and the MIND diet, or adopting a more anti-inflammatory diet (characterised by a lower Dietary Inflammatory Index^®^ (DII), are associated with a reduced risk of depression [6]. Previous research by our group reported associations between a more pro-inflammatory diet and higher likelihood of depressive symptoms, anxiety and poorer well-being [7]. We also recently demonstrated that unhealthy lifestyle behaviours and body mass index (BMI) may largely explain the association between depressive symptoms and low-grade inflammation [8].

Since the publication of the EAT Lancet Commission report [9], there has been increasing interest in plant-based diets (PBDs). Characterised by a high intake of fruits, vegetables, nuts, seeds and whole grains, PBDs align with global recommendations aimed at preventing chronic diseases and enhancing mental health [10]. PBD indices (PDIs) which distinguish between healthy and unhealthy PBDs have also been created, in recognition that not all plant-based dietary components are healthy [11]. However, a limited number of studies examining PDIs and mental health outcomes have been conducted and the majority have focussed on cognitive function and decline, with only a handful of published papers examining depressive symptoms and anxiety [12–15]. In addition, these publications are based on a single population source, and results have been mixed.

A greater understanding of relationships between PBDs and chronic non-communicable disease is important for informing the development of risk mitigation strategies. Therefore, the aim of this study was to investigate an overall PDI (oPDI), healthy PDI (hPDI) and unhealthy PDI (uPDI) associations with depressive symptoms, anxiety and well-being, using a cross-sectional random sample of 1,949 middle- to older-aged adults. We tested the hypothesis that greater adherence to an oPDI and hPDI, and lower adherence to a uPDI, would be associated with better mental health.

## Materials and methods

### Study population and setting

The Cork and Kerry Diabetes and Heart Disease Study (Phase II) was a single centre, cross-sectional study conducted between 2010 and 2011. Full details regarding the study design have been previously described [16]. In brief, the study population were patients that attended the Living Health Clinic in Mitchelstown, County Cork, Ireland. Participants were randomly selected from all registered middle- to older-aged (46–70 years) attending patients and a stratified sample was recruited. Of 3,807 potential participants, following the exclusion of duplicates, deaths and individuals who were incapable of consenting or attending appointment, 3,051 were invited to take part in the study. Of these, 2,047 (49% male) completed the questionnaire and physical examinations of the baseline assessment (response of 67%). The sample was broadly similar to the local background population in terms of proportional age representation, marital status and education, representing a low risk of selection bias.

Ethics committee approval conforming to the Declaration of Helsinki was obtained from the Clinical Research Ethics Committee of University College Cork. All participants provided written informed consent to use their data for research purposes. Dietary data for the current analysis were available for 1,949 subjects.

## Clinical procedures

Study participants attended the clinic in the morning after an overnight fast and blood samples were taken on arrival. Fasting glucose and glycated haemoglobin A_1c_ (HbA_1c_) concentrations were measured in fresh samples by Cork University Hospital Biochemistry Laboratory using standardised procedures. Glucose concentrations were determined using a glucose hexokinase assay (Olympus Life and Material Science Europa Ltd., Lismeehan, Co. Clare, Ireland) and HbA_1c_ levels were measured in the haematology laboratory on an automated high-pressure liquid chromatography instrument Tosoh G7 [Tosoh HLC-723 (G7), Tosoh Europe N.V, Tessenderlo, Belgium]. Clinical measurements were performed by trained researchers with reference to a standard operating procedures manual. Height was measured with a portable Seca Leicester height/length stadiometer (Seca, Birmingham, UK) and weight was measured using a portable electronic Tanita WB-100MA weighing scale (Tanita Corp, IL, USA). The weighing scale was placed on a firm flat surface and was calibrated weekly. BMI (BMI=weight(kg)/height(m)^2^) was calculated from measured weight and height.

## Dietary assessment and plant-based diet indices

A modified version of the self-completed European Prospective Investigation into Cancer and Nutrition (EPIC) food frequency questionnaire (FFQ) [17], which was adapted to reflect the Irish diet, was used to assess dietary intake. This 150-item semi-quantitative FFQ has been validated for use in the Irish population [18]. Further details have been provided elsewhere [5].

PDIs were created using the method described by Satija et al. [11], and described in full here [19]. Briefly, an oPDI, hPDI and uPDI were each created from 18 food groups (Supplementary Table 1). Healthy plant food groups included whole grains, fruits, vegetables, nuts, legumes, vegetable oils and tea/coffee, whereas unhealthy plant food groups included fruit juices, sugar-sweetened beverages (SSBs), refined grains, potatoes and sweets/desserts. Animal food groups included animal fats, dairy, eggs, fish/seafood, poultry/red meat and miscellaneous animal-based foods. Frequencies of food consumption were converted into servings consumed per day. The number of servings of foods that belonged to each food group were then totalled. Food groups were divided into quintiles of consumption, with each scored between 1 and 5, except for nuts, which were divided into tertiles of consumption, scored between 1 and 3, and SSBs and eggs which were divided into quartiles of consumption, scored between 1 and 4. For the oPDI, positive scoring was used for healthy and unhealthy plant foods and reverse scoring was used for animal foods. For the hPDI, positive scoring was given to healthy plant foods only, with reverse scoring for unhealthy plant foods and animal foods. For the uPDI, positive scoring was given to unhealthy plant foods only, with reverse scoring for healthy plant foods and animal foods. The 18 food groups were then summed to give an overall score for the three indices, with a possible range of 18 (lowest) to 86 (highest). Observed ranges in the current study were 31–72 for the oPDI, 31–76 for the hPDI and 30–75 for the uPDI. For the oPDI, higher scores represent a more overall PBD. For the hPDI, higher scores represent a healthier PBD. For the uPDI, higher scores represent an unhealthier PBD.

## Mental health outcomes

Mental health status was evaluated using validated screening tools. Depressive symptoms were assessed using the Center for Epidemiologic Studies Depression (CES-D) Scale, which consists of 20 items scored on a scale from 0 to 3, with higher scores indicating greater depressive symptomatology [20]. Anxiety was measured using the anxiety sub-scale of the Hospital Anxiety and Depression Scale (HADS-A), scored from 0 to 21, with higher scores reflecting greater anxiety [21]. Overall well-being was evaluated using the World Health Organization-Five (WHO-5) Well-Being Index, a 5-item questionnaire, with each item scored from 0 to 5, and total scores ranging from 0 to 25. Higher scores represent greater well-being [22].

## Covariates

A general health and lifestyle questionnaire (GHLQ) assessed demographic variables, lifestyle behaviours, medication use and morbidity. Participants provided information on age, sex, education, smoking status, alcohol intake, anti-depressant use and history of type 2 diabetes, cardiovascular disease and cancer. Physical activity levels were measured using the validated International Physical Activity Questionnaire (IPAQ) [23].

Education was defined as ‘secondary or higher’ or ‘primary level only’. Smoking status was defined as ‘non-smoker’ and ‘current smoker’. Alcohol consumption was measured in units of alcohol consumed on a weekly basis and was categorised into the following levels: (i) non-drinker, i.e. <1 drink per week; (ii) moderate drinker, i.e. between 1 and 14 drinks per week; and (iii) heavy drinker, i.e. >14 drinks per week. Moderate drinker was defined on the basis of previous work from the EPIC in the United Kingdom by Khaw et al. [24]. For the current analysis, these were then re-categorised as ‘moderate/non-drinker’ or ‘heavy drinker’. Physical activity was categorised as high, moderate or low levels of activity using the IPAQ. This was then recoded as a dichotomous variable: ‘moderate/high’ or ‘low’ level physical activity.

Type 2 diabetes was determined as a fasting glucose level *≥* 7.0 mmol/l or HbA_1c_ level *≥* 6.5% (*≥* 48 mmol/mol) [25] or by self-reported diagnosis. The presence of cardiovascular disease was obtained by asking study participants if they had been diagnosed with any one of the following seven conditions: Heart Attack (including coronary thrombosis or myocardial infarction), Heart Failure, Angina, Aortic Aneurysm, Hardening of the Arteries, Stroke or any other Heart Trouble. Subjects who indicated a diagnosis of any one of these conditions were classified as having cardiovascular disease. Cancer was defined as a binary (yes/no) variable as determined from participants’ self-reported responses to the GHLQ.

### Statistical analysis

Participant characteristics, mental health scores and dietary intake were examined according to oPDI, hPDI and uPDI quartiles Categorical features are presented as numbers (n) and percentages (%) and continuous variables are shown as a mean, plus or minus one standard deviation (± SD), or a median and interquartile range (IQR) for skewed data. Differences were analysed using a Pearson’s chi-square test, one-way ANOVA or a Kruskal-Wallis test. Correlations between PDIs and mental health scores were assessed using Spearman’s rank-order correlation. Linear regression analysis investigated oPDI, hPDI and uPDI score (continuous and quartile) associations with depressive symptoms, anxiety and well-being (outcome variables). Four models were run; Model 1 was age and sex-adjusted, while Model 2 additionally adjusted for energy intake, BMI and anti-depressant use. Model 3 also adjusted for education and lifestyle behaviours (smoking, alcohol use and physical activity). A final model additionally adjusted for history of type 2 diabetes, cardiovascular disease and cancer. When analyses were performed, the assumptions of linear regression were checked, and the expectations of heteroscedasticity and normality of the residuals were met. Logistic regression analyses were also performed using a CES-D cut-off score of *≥* 16 for clinical depression (*n* = 297). Data analyses were conducted using Stata SE Version 13 (Stata Corporation, College Station, TX, USA) for Windows. For all analyses, a *p* value (two-tailed) of less than 0.05 was considered to indicate statistical significance.

## Results

### Participant characteristics according to oPDI, hPDI and uPDI quartiles

Examination of participant characteristics according to quartiles of each PDI (Table 1) revealed sex differences and that those with higher oPDI scores were younger while those with higher hPDI scores were older. Higher oPDI and hPDI scores were also associated with a having a lower BMI. Participants in the highest oPDI quartile had a higher educational attainment compared to the lowest quartile, while subjects in the highest uPDI quartile were more likely to have been educated to a primary level only. With regard to lifestyle behaviours, participants in the highest oPDI quartile were less likely to be heavy drinkers, while those in the highest hPDI quartile were less likely to be current smokers or to have low levels of physical activity. Those in the highest uPDI quartile were more likely to be current smokers, heavy drinkers and had lower levels of physical activity. Regarding mental health scores, there was a trend towards less depressive symptoms (as indicated by lower CES-D scores) among those with greater adherence to the oPDI (*p* = 0.029) and hPDI (*p* = 0.002). Subjects in the highest hPDI quartile also displayed greater well-being as indicated by higher WHO-5 scores (*p* = 0.028). With regard to PDIs, mean hPDI scores significantly increased across oPDI quartiles, while uPDI scores significantly decreased across hPDI quartiles.

**Table 1:** Characteristics of the study participants according to oPDI, hPDI and uPDI score quartiles

*n*	oPDI Q1	oPDI Q2	oPDI Q3	oPDI Q4	*p*
	535	507	461	446	
Age in years (median, IQR)	60.2 (55.4, 64.9)	59.5 (55.1, 64.1)	59.5 (54.9, 63.6)	58.5 (54.4, 63.5)	**0.024**
Male (n, %)	299 (55.9)	239 (47.1)	207 (44.9)	210 (47.1)	**0.002**
BMI, kg/m² (mean ± SD)	29.3 ± 4.7	28.3 ± 4.7	28.5 ± 4.9	28.0 ± 4.4	**< 0.001**
Anti-depressant use (n, %)	25 (4.7)	19 (3.7)	21 (4.6)	25 (5.6)	0.6
Primary education only (n, %)	172 (32.1)	128 (25.2)	109 (23.6)	103 (23.1)	**0.003**
Current smoker (n, %)	84 (15.7)	73 (14.4)	67 (14.5)	55 (12.3)	0.515
Heavy drinker (n, %)	79 (14.8)	45 (8.9)	30 (6.5)	35 (7.8)	**< 0.001**
Low-level physical activity (n, %)	253 (47.3)	234 (46.2)	210 (45.6)	197 (44.2)	0.804
Type 2 diabetes (n, %)	65 (12.1)	50 (9.9)	29 (6.3)	28 (6.3)	**0.002**
Cardiovascular disease (n, %)	66 (12.3)	56 (11.0)	40 (8.7)	41 (9.2)	0.209
Cancer (n, %)	24 (4.5)	23 (4.5)	14 (3.0)	16 (3.6)	0.566
CES-D score (median, IQR)	9.0 (4.0, 14.0)	7.0 (3.0, 12.0)	7.0 (4.0, 12.0)	7.0 (3.0, 12.0)	**0.029**
HADS-A score (median, IQR)	3.0 (2.0, 6.0)	4.0 (2.0, 6.0)	4.0 (2.0, 6.0)	4.0 (2.0, 6.0)	0.885
WHO-5 score (median, IQR)	18.0 (14.0, 20.0)	18.0 (14.0, 20.0)	18.0 (14.0, 20.0)	18.0 (14.0, 20.0)	0.481
oPDI (mean ± SD)	43.9 ± 2.8	49.6 ± 1.1	53.5 ± 1.1	59.2 ± 2.9	**< 0.001**
hPDI (mean ± SD)	50.5 ± 6.2	52.6 ± 6.8	53.6 ± 7.6	55.0 ± 6.9	**< 0.001**
uPDI (mean ± SD)	52.4 ± 7.2	51.7 ± 7.1	51.5 ± 7.4	52.3 ± 7.0	0.176
	**hPDI Q1**	**hPDI Q2**	**hPDI Q3**	**hPDI Q4**	***p***
n	563	487	477	422	
Age in years (median, IQR)	58.0 (54.1, 63.4)	59.5 (54.9, 64.0)	60.2 (55.2, 64.5)	60.5 (56.0, 64.9)	**< 0.001**
Male (n, %)	325 (57.5)	255 (52.4)	208 (43.6)	167 (39.6)	**< 0.001**
BMI, kg/m² (mean ± SD)	29.1 ± 4.8	28.9 ± 4.7	28.3 ± 4.8	27.8 ± 4.4	**< 0.001**
Anti-depressant use (n, %)	25 (4.4)	30 (6.2)	22 (4.6)	13 (3.1)	0.177
Primary education only (n, %)	142 (25.2)	123 (25.3)	134 (28.1)	113 (26.8)	0.694
Current smoker (n, %)	102 (18.1)	80 (16.4)	51 (10.7)	46 (10.9)	**0.001**
Heavy drinker (n, %)	59 (10.5)	56 (11.5)	40 (8.4)	34 (8.1)	0.218
Low-level physical activity (n, %)	284 (50.5)	222 (45.6)	213 (44.7)	175 (41.5)	**0.04**
Type 2 diabetes (n, %)	56 (9.9)	40 (8.2)	38 (8.0)	38 (9.0)	0.668
Cardiovascular disease (n, %)	63 (11.2)	46 (9.4)	50 (10.5)	44 (10.4)	0.836
Cancer (n, %)	23 (4.1)	14 (2.9)	22 (4.6)	18 (4.3)	0.537
CES-D score (median, IQR)	8.0 (4.8, 13.0)	8.0 (3.0, 14.0)	7.5 (4.0, 12.0)	7.0 (3.0, 12.0)	**0.002**
HADS-A score (median, IQR)	4.0 (2.0, 6.0)	4.0 (2.0, 6.0)	4.0 (2.0, 6.0)	3.0 (1.0, 6.0)	0.466
WHO-5 score (median, IQR)	18.0 (13.0, 20.0)	18.0 (14.0, 20.0)	18.0 (14.0, 20.0)	19.0 (14.0, 20.0)	**0.028**
oPDI (mean ± SD)	49.5 ± 5.6	50.5 ± 6.0	51.8 ± 6.1	53.4 ± 5.5	**< 0.001**
hPDI (mean ± SD)	44.4 ± 3.2	51.0 ± 1.4	55.9 ± 1.4	62.5 ± 3.2	**< 0.001**
uPDI (mean ± SD)	53.7 ± 6.9	52.4 ± 7.5	51.3 ± 7.1	50.0 ± 6.5	**< 0.001**
	**uPDI Q1**	**uPDI Q2**	**uPDI Q3**	**uPDI Q4**	***p***
n	526	538	441	444	
Age in years (median, IQR)	58.8 (54.6, 63.5)	59.3 (55.0, 63.9)	59.6 (55.0, 64.6)	60.2 (55.3, 64.6)	0.163
Male (n, %)	197 (37.5)	248 (46.1)	247 (56.0)	263 (59.2)	**< 0.001**
BMI, kg/m² (mean ± SD)	28.3 ± 4.8	28.8 ± 4.8	28.5 ± 4.5	28.6 ± 4.7	0.353
Anti-depressant use (n, %)	15 (2.9)	32 (5.9)	25 (5.7)	18 (4.1)	0.063
Primary education only (n, %)	101 (19.2)	121 (22.5)	125 (28.3)	165 (37.2)	**< 0.001**
Current smoker (n, %)	55 (10.5)	76 (14.1)	76 (17.2)	72 (16.2)	**0.013**
Heavy drinker (n, %)	39 (7.4)	50 (9.3)	58 (13.2)	42 (9.5)	**0.026**
Low-level physical activity (n, %)	221 (42.0)	221 (41.1)	212 (48.1)	240 (54.1)	**< 0.001**
Type 2 diabetes (n, %)	47 (8.9)	51 (9.5)	28 (6.3)	46 (10.4)	0.175
Cardiovascular disease (n, %)	50 (9.5)	58 (10.8)	43 (9.8)	52 (11.7)	0.67
Cancer (n, %)	24 (4.6)	22 (4.1)	11 (2.5)	20 (4.5)	0.338
CES-D score (median, IQR)	7.0 (4.0, 12.0)	8.0 (3.0, 13.0)	7.0 (4.0, 12.8)	8.0 (4.0, 13.0)	0.817
HADS-A score (median, IQR)	4.0 (2.0, 6.0)	4.0 (2.0, 6.0)	4.0 (2.0, 6.0)	3.0 (1.0, 6.0)	0.145
WHO-5 score (median, IQR)	18.0 (14.0, 20.0)	18.0 (14.0, 20.0)	18.0 (14.0, 20.0)	18.0 (14.0, 20.0)	0.795
oPDI (mean ± SD)	51.2 ± 5.6	51.3 ± 5.9	51.1 ± 6.3	51.0 ± 6.1	0.874
hPDI (mean ± SD)	54.7 ± 6.9	53.0 ± 7.0	52.3 ± 7.4	50.7 ± 6.3	**< 0.001**
uPDI (mean ± SD)	43.4 ± 3.3	50.0 ± 1.4	54.9 ± 1.4	61.8 ± 3.3	**< 0.001**

CES-D: Center for Epidemiologic Studies Depression; HADS-A: Hospital Anxiety and Depression Scale-Anxiety; WHO-5: World Health Organization-Five Well-Being Index. oPDI: Overall Plant-Based Diet Index; hPDI: Healthy Plant-Based Diet Index; uPDI: Unhealthy Plant-Based Diet Index. Categorical variables are presented as numbers (n) and percentages (%). Continuous values are presented as a mean ± one standard deviation (SD) or a median with interquartile range (IQR). The *p* values were determined from a chi-square test, one-way ANOVA or a Kruskal-Wallis test. Significant *p* shown in bold.

Participants’ dietary intake across quartiles of the oPDI, hPDI and uPDI are shown in Table 2. Compared to those in the lowest quartile of the oPDI, total energy intake and absolute dietary macronutrient intake (grams/day) were consistently higher in subjects with greater adherence to the oPDI. In contrast, higher hPDI and uPDI quartiles were associated with lower energy intake and lower consumption of fats, carbohydrates, protein and sugar. Results were mostly similar when examined as contribution to energy intake, with some exceptions. The contribution of fats and protein to total energy intake decreased across oPDI quartiles, while the contribution of carbohydrates (including sugar and fibre) and protein increased across hPDI quartiles; the contribution of carbohydrates to total energy intake also increased across uPDI quartiles.

**Table 2:** Nutritional intake of the study population according to oPDI, hPDI and uPDI score quartiles

Dietary composition	oPDI Q1	oPDI Q2	oPDI Q3	oPDI Q4	*p*
Energy intake, kcal (mean ± SD)	1731.7 ± 690.7	1957.9 ± 754.6	2132.7 ± 800.5	2418.7 ± 847.5	**< 0.001**
Fat, g/d (mean ± SD)	69.7 ± 32.8	75. 2 ± 36.1	81.2 ± 40.5	86.2 ± 40.1	**< 0.001**
SFA, g/d (mean ± SD)	25.3 ± 13.9	26.7 ± 14.7	28.1 ± 15.4	28.8 ± 14.9	**0.001**
PUFA, g/d (mean ± SD)	13.3 ± 7.3	14.7 ± 8.3	16.7 ± 11.0	18.0 ± 10.1	**< 0.001**
MUFA, g/d (mean ± SD)	22.1 ± 10.3	23.8 ± 11.6	25.4 ± 12.8	26.9 ± 13.1	**< 0.001**
Carbohydrate, g/d (mean ± SD)	191.7 ± 84.8	235.4 ± 98.1	266.2 ± 91.1	323.7 ± 115.7	**< 0.01**
Protein, g/d (mean ± SD)	85.6 ± 36.3	91.1 ± 38.7	93.9 ± 38.9	99.0 ± 34.1	**< 0.001**
Sugar, g/d (mean ± SD)	76.9 ± 48.2	100.1 ± 57.3	115.0 ± 54.0	138.1 ± 60.5	**< 0.001**
Fibre, g/d (mean ± SD)	19.7 ± 10.2	24.6 ± 10.4	27.8 ± 10.4	34.1 ± 13.0	**< 0.001**
*% of energy intake*					
Fat, % (mean ± SD)	35.9 ± 6.9	34.0 ± 6.5	33.4 ± 6.9	31.6 ± 6.2	**< 0.001**
SFA, % (mean ± SD)	12.9 ± 3.8	12.0 ± 3.5	11.5 ± 3.3	10.5 ± 2.9	**< 0.001**
PUFA, % (mean ± SD)	6.9 ± 2.3	6.6 ± 2.1	6.8 ± 2.5	6.6 ± 2.3	0.155
MUFA, % (mean ± SD)	11.4 ± 2.3	10.8 ± 2.2	10.5 ± 2.3	9.8 ± 2.2	**< 0.001**
Carbohydrate, % (mean ± SD)	44.3 ± 8.1	48.3 ± 7.3	50.6 ± 7.2	53.8 ± 7.1	**< 0.001**
Protein, % (mean ± SD)	20.2 ± 4.3	18.9 ± 4.0	17.9 ± 3.4	16.6 ± 3.0	**< 0.001**
Sugar, % (mean ± SD)	17.8 ± 6.9	20.5 ± 7.8	21.8 ± 6.7	23.1 ± 6.6	**< 0.001**
Fibre, % (mean ± SD)	2.3 ± 0.8	2.6 ± 0.7	2.7 ± 0.7	2.9 ± 0.6	**< 0.001**
*% of total fat*					
SFA, % (mean ± SD)	35.7 ± 6.2	35.0 ± 6.1	34.2 ± 6.0	33.1 ± 6.1	**< 0.001**
PUFA, % (mean ± SD)	19.3 ± 5.5	19.7 ± 5.5	20.3 ± 5.7	20.8 ± 5.7	**< 0.001**
MUFA, % (mean ± SD)	31.9 ± 3.1	31.8 ± 3.2	31.4 ± 3.3	31.1 ± 3.1	**< 0.001**
*Dietary composition*	**hPDI Q1**	**hPDI Q2**	**hPDI Q3**	**hPDI Q4**	***p***
Energy intake, kcal (mean ± SD)	2458.4 ± 880.4	2070.4 ± 754.0	1869.1 ± 688.8	1651.9 ± 626.4	**< 0.001**
Fat, g/d (mean ± SD)	100.1 ± 42.1	79.0 ± 34.7	68.0 ± 30.0	57.0 ± 24.7	**< 0.001**
SFA, g/d (mean ± SD)	36.8 ± 16.1	27.9 ± 13.9	22.8 ± 11.1	18.3 ± 8.6	**< 0.001**
PUFA, g/d (mean ± SD)	19.7 ± 10.7	16.1 ± 88.1	13.9 ± 8.0	11.4 ± 6.0	**< 0.001**
MUFA, g/d (mean ± SD)	31.4 ± 13.5	24.8 ± 10.6	21.5 ± 9.8	17.9 ± 8.6	**< 0.001**
Carbohydrate, g/d (mean ± SD)	291.1 ± 116.2	253.0 ± 105.7	235.3 ± 104.2	212.5 ± 92.8	**< 0.001**
Protein, g/d (mean ± SD)	106.9 ± 39.3	93.3 ± 35.0	85.8 ± 34.0	78.0 ± 33.6	**< 0.001**
Sugar, g/d (mean ± SD)	119.4 ± 59.3	104.2 ± 57.0	102.4 ± 64.9	94.1 ± 51.3	**< 0.001**
Fibre, g/d (mean ± SD)	26.3 ± 11.4	26.1 ± 13.0	26.0 ± 12.6	26.5 ± 11.8	0.938
*% of energy intake*					
Fat, % (mean ± SD)	36.3 ± 5.9	34.2 ± 6.8	32.6 ± 6.7	31.3 ± 6.8	**< 0.001**
SFA, % (mean ± SD)	13.4 ± 3.4	12.1 ± 3.5	11.0 ± 3.3	10.1 ± 2.9	**< 0.001**
PUFA, % (mean ± SD)	7.0 ± 2.2	6.9 ± 2.3	6.6 ± 2.4	6.2 ± 2.3	**< 0.001**
MUFA, % (mean ± SD)	11.4 ± 2.0	10.8 ± 2.2	10.3 ± 2.4	9.8 ± 2.5	**< 0.001**
Carbohydrate, % (mean ± SD)	47.2 ± 7.3	48.6 ± 8.3	49.9 ± 8.4	50.8 ± 8.7	**< 0.001**
Protein, % (mean ± SD)	17.7 ± 3.4	18.4 ± 3.7	18.8 ± 4.1	19.3 ± 4.7	**< 0.001**
Sugar, % (mean ± SD)	19.3 ± 6.0	19.9 ± 7.0	21.3 ± 7.6	22.5 ± 8.4	**< 0.001**
Fibre, % (mean ± SD)	2.1 ± 0.5	2.5 ± 0.6	2.8 ± 0.7	3.2 ± 0.8	**< 0.001**
*% of total fat*					
SFA, % (mean ± SD)	36.8 ± 5.8	35.1 ± 6.0	33.5 ± 6.1	32.1 ± 5.7	**< 0.001**
PUFA, % (mean ± SD)	19.4 ± 5.2	20.3 ± 5.6	20.4 ± 5.8	19.9 ± 5.9	**0.025**
MUFA, % (mean ± SD)	31.5 ± 2.6	31.7 ± 2.9	31.7 ± 2.9	31.4 ± 4.0	0.377
*Dietary composition*	**uPDI Q1**	**uPDI Q2**	**uPDI Q3**	**uPDI Q4**	***p***
Energy intake, kcal (mean ± SD)	2437.4 ± 880.4	2127.6 ± 755.7	1904.6 ± 689.5	1609.1 ± 635.5	**< 0.001**
Fat, g/d (mean ± SD)	94.2 ± 42.2	81.7 ± 36.8	72.1 ± 30.8	58.6 ± 28.6	**< 0.001**
SFA, g/d (mean ± SD)	31.8 ± 16.0	28.6 ± 14.9	25.5 ± 12.7	21.5 ± 12.8	**< 0.001**
PUFA, g/d (mean ± SD)	19.2 ± 10.1	16.5 ± 9.8	14.4 ± 8.5	11.2 ± 6.3	**< 0.001**
MUFA, g/d (mean ± SD)	29.8 ± 14.2	25.5 ± 11.3	22.4 ± 9.5	18.6 ± 8.9	**< 0.001**
Carbohydrate, g/d (mean ± SD)	291.5 ± 113.8	260.3 ± 107.2	235.7 ± 102.9	206.5 ± 94.4	**< 0.001**
Protein, g/d (mean ± SD)	115.6 ± 46.0	95.5 ± 28.7	83.5 ± 26.7	68.5 ± 24.8	**< 0.001**
Sugar, g/d (mean ± SD)	130.7 ± 65.2	109.2 ± 58.1	96.4 ± 53.3	82.2 ± 45.6	**< 0.001**
Fibre, g/d (mean ± SD)	33.9 ± 12.7	27.8 ± 11.4	23.3 ± 9.6	18.1 ± 8.1	**< 0.001**
*% of energy intake*					
Fat, % (mean ± SD)	34.3 ± 6.3	34.1 ± 6.7	34.0 ± 6.8	32.6 ± 7.4	**< 0.001**
SFA, % (mean ± SD)	11.5 ± 3.2	11.8 ± 3.5	11.9 ± 3.4	11.8 ± 4.1	0.343
PUFA, % (mean ± SD)	7.0 ± 2.1	6.8 ± 2.3	6.8 ± 2.6	6.2 ± 2.1	**< 0.001**
MUFA, % (mean ± SD)	10.8 ± 2.3	10.7 ± 2.3	10.6 ± 2.2	10.4 ± 2.4	**0.02**
Carbohydrate, % (mean ± SD)	47.9 ± 7.3	48.6 ± 7.5	48.9 ± 8.3	50.8 ± 9.6	**< 0.001**
Protein, % (mean ± SD)	19.3 ± 3.8	18.6 ± 3.8	18.2 ± 3.9	17.7 ± 4.3	**< 0.001**
Sugar, % EI (mean ± SD)	21.6 ± 7.1	20.5 ± 7.2	20.0 ± 6.9	20.4 ± 8.0	**0.004**
Fibre, % EI (mean ± SD)	2.9 ± 0.7	2.7 ± 0.7	2.5 ± 0.7	2.3 ± 0.7	**< 0.001**
*% of total fat*					
SFA, % (mean ± SD)	33.4 ± 5.5	34.4 ± 6.0	34.8 ± 6.4	35.8 ± 6.7	**< 0.001**
PUFA, % (mean ± SD)	20.4 ± 5.1	20.1 ± 5.5	19.9 ± 6.0	19.4 ± 5.9	**0.04**
MUFA, % (mean ± SD)	31.5 ± 3.2	31.5 ± 3.1	31.3 ± 3.2	32.0 ± 3.3	**0.012**

MUFA: monounsaturated fatty acids; PUFA: polyunsaturated fatty acids; SFA: saturated fatty acids. oPDI: Overall Plant-Based Diet Index; hPDI: Healthy Plant-Based Diet Index; uPDI: Unhealthy Plant-Based Diet Index. Values are presented as a mean ± one standard deviation (SD). The *p* values were determined from a one-way ANOVA. Significant *p* shown in bold.

### Correlation analyses

A correlation matrix of PDIs and mental health scores is shown in Table 3. Weak, but significant inverse correlations were observed between CES-D scores and the oPDI (ρ = -0.067; *p* < 0.01) and hPDI (ρ = -0.082; *p* < 0.001). The hPDI was also positively correlated with the WHO-5 score (ρ = 0.073; *p* < 0.01). All mental health scores were significantly correlated, with the CES-D score being positively correlated with the HADS-A score (ρ = 0.605; *p* < 0.001), while the WHO-5 score was inversely correlated with both the CES-D and HADS-A scores (ρ = -0.511; *p* < 0.001 and ρ = -0.505; *p* < 0.001).

**Table 3:** Correlation matrix of plant-based dietary indices and mental health scores

hPDI	oPDI	hPDI	uPDI	CES-D score	HADS-A score
	0.238**				
uPDI	−0.017	−0.201**			
CES-D score	−0.067*	−0.082**	0.010		
HADS-A score	−0.014	−0.034	−0.064	0.605**	
WHO-5 score	0.019	0.073*	0.012	−0.511**	−0.505**

CES-D: Center for Epidemiologic Studies Depression; HADS-A: Hospital Anxiety and Depression Scale-Anxiety; WHO-5: World Health Organization-Five Well-Being Index. oPDI: Overall Plant-Based Diet Index; hPDI: Healthy Plant-Based Diet Index; uPDI: Unhealthy Plant-Based Diet Index. Values are presented as Spearman correlation coefficients. Significant correlation coefficient values shown in bold. *p < 0.01; **p < 0.001

### Linear regression

**Fig. 1:**
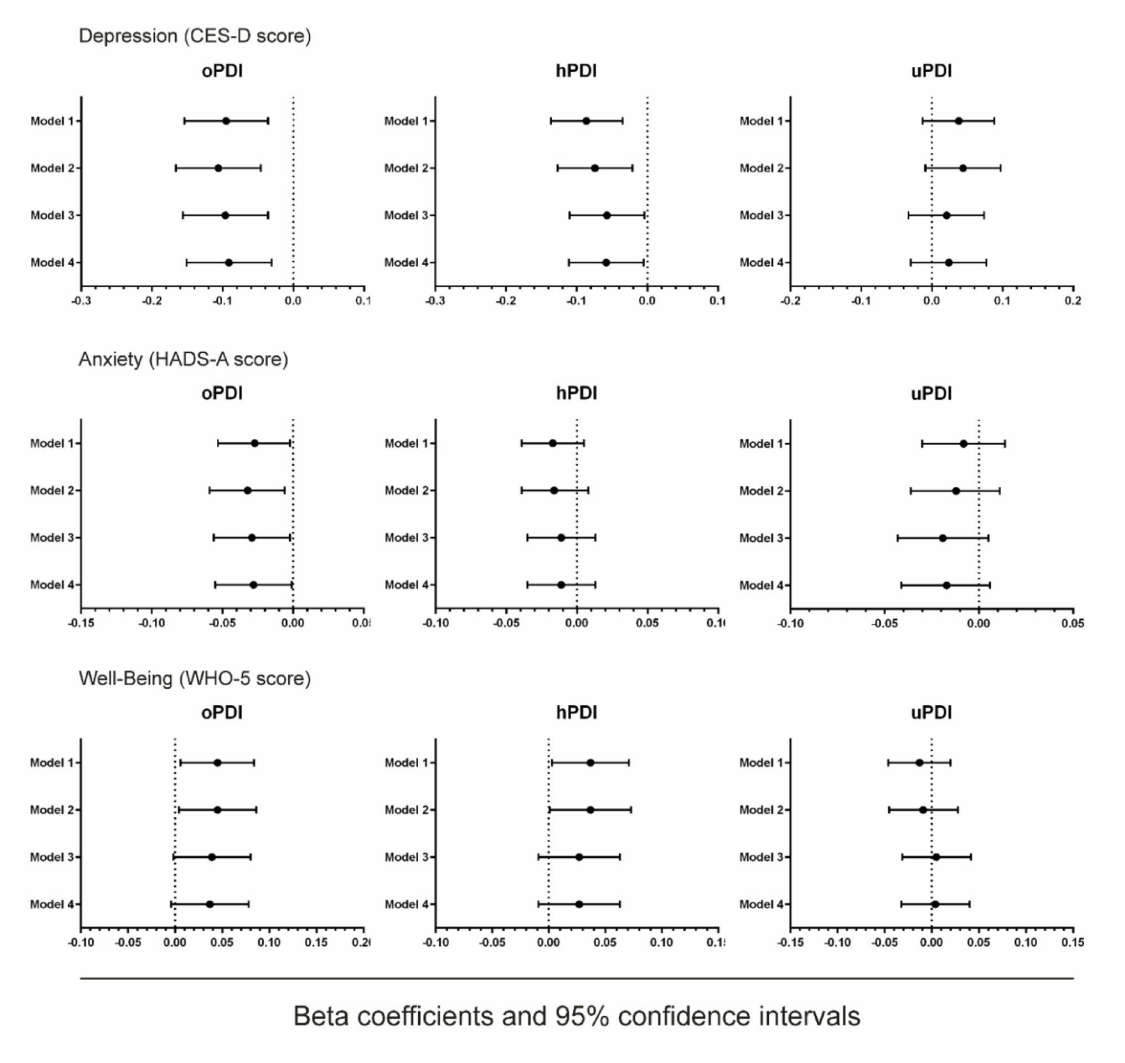
Linear regression analysis of oPDI, hPDI and uPDI associations with mental health scores. Beta coefficients and 95% confidence intervals are shown. Model 1: adjusted for age and sex. Model 2: adjusted for age, sex, energy intake, BMI and anti-depressant use. Model 3: adjusted for age, sex, energy intake, BMI, anti-depressant use, education, smoking, alcohol use and physical activity. Model 4: additionally adjusted for history of type 2 diabetes, cardiovascular disease and cancer. CES-D: Center for Epidemiologic Studies Depression; HADS-A: Hospital Anxiety and Depression Scale-Anxiety; WHO-5: World Health Organization-Five Well-Being Index. oPDI: Overall Plant-Based Diet Index; hPDI: Healthy Plant-Based Diet Index; uPDI: Unhealthy Plant-Based Diet Index.

The results of linear regression analyses which examined associations between PDIs and each of the mental health scores are presented in Fig. 1. In age and sex-adjusted models, the oPDI and hPDI were negatively associated with depressive symptoms (β = -0.095, 95% CI: -0.154, -0.036; *p* = 0.002 and β = -0.086, 95% CI: -0.136, -0.035; *p* = 0.001, for oPDI and hPDI scores respectively), and positively associated with greater well-being (β = 0.045, 95% CI: 0.006, 0.084; *p* = 0.025 and β = 0.037, 95% CI: 0.003, 0.071; *p* = 0.032, for oPDI and hPDI scores respectively). Additionally, the oPDI was inversely associated with anxiety (β = -0.027, 95% CI: -0.053, -0.002; *p* = 0.038). In models which also adjusted for energy intake, BMI, anti-depressant medication use, education, lifestyle factors and morbidity (Model 4), the oPDI demonstrated a significant inverse association with higher HADS-A scores (β = -0.028, 95% CI: -0.055, -0.001; *p* = 0.043). Significant inverse relationships between the oPDI, hPDI and depressive symptoms also persisted upon full adjustment, whereby a 1 unit increase in the oPDI and hPDI was associated with a corresponding 0.09 (β = -0.091, 95% CI: -0.151, -0.031; *p* = 0.003) and 0.06 (β = -0.058, 95% CI: -0.111, -0.005; *p* = 0.031) unit decrease in the CES-D score. Furthermore, associations between oPDI and hPDI quartiles and CES-D scores (Supplementary Table 2) demonstrated a significant dose-response relationship (*p* trend < 0.05). No associations between the uPDI and mental health scores were observed in any model.

### Logistic regression

As both the oPDI and hPDI displayed significant relationships with CES-D scores in linear regression models when examined as continuous variables, and as quartiles, we additionally conducted logistic regression analyses using a CES-D clinical cut-off score of *≥* 16 (*n* = 297), the results of which are presented in Supplementary Table 3. Although participants in the lowest quartiles of the oPDI and hPDI displayed a greater risk of depression when compared to those in the highest quartile, these relationships were not statistically significant in fully adjusted models, with confidence intervals for effect estimates including the value 1. In addition, although age and sex-adjusted models suggested a dose-response relationship between hPDI quartiles and depression (*p* trend = 0.02), this association was attenuated in models which adjusted for potential confounders.

## Discussion

This research aimed to explore PBD associations with self-reported depressive symptoms, anxiety and well-being among a population of middle- to older-aged adults. In agreement with our hypothesis, these findings show that greater adherence to a more overall PBD (characterised by higher oPDI scores) was associated with less depressive symptoms, less anxiety and greater well-being. A more healthful PBD (characterised by higher hPDI scores) was associated with lower depressive symptoms and better well-being. In fully adjusted models, oPDI and hPDI associations with depressive symptoms, and oPDI associations with anxiety, persisted. However, the uPDI did not show significant associations with any of the mental health scores.

The oPDI and hPDI results are consistent with previous research, including our own, which suggest that diets rich in fruits, vegetables, whole grains, nuts and legumes are beneficial for mental health. In a meta-analysis of eight cross-sectional studies that met inclusion criteria, Shafiei et al. found a significant inverse association between adherence to the Mediterranean diet and depression: overall odds ratio (OR) = 0.81, 95% CI: 0.71, 0.92. When effect sizes obtained from three cohort studies were examined, a significant inverse association between the Mediterranean diet and depression was also observed: overall hazard ratio = 0.81, 95% CI: 0.70, 0.94 [26]. Research conducted by our group has demonstrated a more pro-inflammatory diet (assessed by the DII) to be associated with higher likelihood of depressive symptoms, anxiety and poorer well-being [7]. We have also previously highlighted better dietary quality (determined by higher Dietary Approaches to Stop Hypertension (DASH) scores) to be associated with greater well-being [27]. A systematic review and meta-analysis of healthy diet scores and depression additionally concluded that greater adherence to a Mediterranean diet, and having a more anti-inflammatory diet, was linked with a lower likelihood of depressive symptoms and a reduced risk of clinical depression [6]. Importantly, in the context of PDIs, previous research by our group demonstrated oPDI and hPDI scores to be negatively correlated with the DII (ρ = -0.288 and ρ = -0.588, respectively) and positively correlated with the DASH score (ρ = 0.231 and ρ = 0.588, respectively) [28].

Despite increasing interest in the health benefits of a PBD, thus far PDIs have been under-researched in the context of mental health, with most research focused on cardiometabolic health outcomes [10, 19, 28, 29]. Of the few studies that have examined PDI associations with psychological health [12–15], results have been inconsistent, perhaps due to the relatively small sample size of some of the studies. Furthermore, they have been based on a single population source (Iran), which limits the generalisability of the findings. Thus, our results represent a novel contribution to the knowledge base.

With regard to these studies, in agreement with our findings that higher oPDI and hPDI scores were associated with less depressive symptoms, in a cross-sectional study of 3,362 subjects, Mousavi et al. reported a lower likelihood of depression among Iranian men and women in the highest oPDI and hPDI quartiles when compared to those in the lowest quartiles (OR = 0.61, 95% CI: 0.44, 0.84 and OR = 0.40, 95% CI: 0.30, 0.53; Q4 vs. Q1 for oPDI and hPDI scores, respectively). They also found a lower likelihood of anxiety among participants with the highest oPDI and hPDI scores (OR = 0.65, 95% CI: 0.43, 0.99 and OR = 0.44, 95% CI: 0.30–0.65; Q4 vs. Q1 for oPDI and hPDI scores, respectively) [14]. In contrast, in a cross-sectional study of 2,033 participants in Iran, Haghighatdoost et al. did not find oPDI or hPDI associations with risk of depression or anxiety [13]. Differences in the instruments used to assess depressive symptoms may partly account for these disparities. For example, we used the 20 item CES-D whereas the HADS instrument was used in the aforementioned Iranian studies.

In the current study we did not find the uPDI to be associated with any of the mental health outcomes. In contrast, Mousavi et al. reported that higher uPDI scores were linked to a greater likelihood of anxiety [14] and Haghighatdoost reported uPDI associations with greater risk of depression and anxiety ([13]. Our research showed only oPDI scores to be inversely associated with a higher risk of anxiety. We also examined PDI associations with psychological well-being (according to WHO-5 scores), to our knowledge for the first time in this context. We found both oPDI and hPDI scores to be positively associated with well-being in age and sex-adjusted models; however, these associations did not remain significant upon full adjustment. While Mousavi et al. reported higher hPDI scores to be associated with a lower likelihood of psychological distress [14], these are not equivalent outcomes.

Previous research investigating a high-quality PBD demonstrated a reduced risk of depression among Australian vegans and vegetarians [30]. Importantly, as reported in that study and others [13, 14, 31], our research shows that following a PBD does not necessarily mean that an individual consumes a ‘healthy’ diet. Although we found greater oPDI and hPDI adherence to be associated with lower contribution of total and saturated fats to total energy intake, the relative contribution of carbohydrates, including sugar, was higher. Interestingly, however, both the oPDI and hPDI demonstrated the same direction of association with mental health scores. In addition, both oPDI and hPDI quartiles showed significant inverse dose-response relationships with depressive symptoms, as indicated by higher CES-D scores.

Collectively, these findings highlight the importance of characterising PBDs and suggest that the overall quality of diet, which reflects the sum of its parts rather than isolated nutrients or food groups, may be important for optimising mental health and psychological well-being. This distinction provides a nuanced view, which is timely considering the EAT-Lancet Commission’s recommendations for a predominantly PBD rich in diverse, healthful foods, benefiting human and planetary health and environmental sustainability [9]. The Planetary Health Diet Index (PHDI) has been developed to assess adherence to the EAT-Lancet recommendations [32]. Recent findings from the PERSIAN Organizational Cohort Study in Mashhad provide novel evidence that higher (Q4) PHDI scores were associated with a 35% reduced risk of depression compared to those in the lowest PHDI quartile [33].

Regarding the mechanisms linking diet and mental health, it is important to consider the complex interactions between nutrients and underlying biological processes. We have recently demonstrated higher uPDI score associations with a lower likelihood of favourable metabolic health status among adults living with and without obesity [34]. Nutrients such as omega-3 fatty acids, folate and other B vitamins have been shown to have neuroprotective effects, supporting brain function and reducing systemic inflammation, which has been linked to mental disorders [35]. The benefits of these nutrients when consumed as part of a healthful dietary pattern highlight the importance of dietary synergy over isolated nutrient supplementation. It should be noted that dietary patterns also influence weight gain, and higher adiposity is strongly associated with a less favourable inflammatory biomarker profile [36, 37]. Our group has shown higher adiposity levels (defined using BMI and waist-height ratio) to be associated with greater depressive symptoms [38], and we recently reported that in addition to diet, unhealthy lifestyle behaviours (smoking, alcohol use, low-level physical activity) and BMI may largely explain the association between depressive symptoms and low-grade inflammation [8]. In the current work we adjust for lifestyle behaviours and BMI in our statistical models. As already mentioned, differences exist across studies which examine PDIs and mental health; for example, BMI was not adjusted for in the Haghighatdoost study [13]. This may partly account for differences seen in our work when compared to findings from previous research.

This study has several strengths including a relatively large, well-characterised cohort and the use of validated diet and mental health assessment instruments, which enhance the reliability of our findings. We also adjusted for a comprehensive range of potential confounders in our statistical models, including demographic, clinical and lifestyle factors, and morbidity, thus providing a robust analysis of the relationship between PBDs and mental health, which strengthen the validity of the findings. However, it is important to consider these results within the limitations of the work. The cross-sectional study design limits our ability to examine temporality of associations and infer causality. Importantly, poorer mental health may adversely affect food choices. Therefore, patients with mental health disorders may find it difficult to follow a PBD [13]. Also, dietary assessment at one time point does not account for changes in diet over time, which could affect the observed associations.

Additionally, dietary data were self-reported, which may introduce recall bias and inaccuracies in reporting. Classification of food items, in terms of their healthfulness, was based on current evidence. However, for some food items assumptions were made which may limit objectivity and introduce subjectivity. Residual confounding is also a possibility. In addition, the study population may limit generalisability of findings to other populations, age groups or ethnicities. It should also be noted that we did not find significant associations in the fully adjusted models between oPDI and hPDI quartiles and depression using a CES-D clinical cut-off score of *≥* 16. While these categorisations are not uncommon, there is a loss of statistical power to detect relationships associated with their use. Nevertheless, it should also be recognised that significant associations between PDIs and mental health scores were modest in our sample. However, this may be partly due to the fact that the diets of individuals in our study were quite similar/monotonous, highlighting the need to examine populations with more diverse diets.

In conclusion, the results from this research suggest that greater adherence to an overall PBD, and more healthful PBD, is associated with fewer depressive symptoms and greater well-being among middle- to older-aged adults. Future longitudinal studies which explore causal relationships between PDIs and psychological outcomes, with a view to the development of targeted dietary recommendations for mental health promotion, are warranted.

## Supplementary Information

Below is the link to the electronic supplementary material.

Supplementary Material 1

## Data Availability

The data used and analysed for the purpose of this study are available from the corresponding author on reasonable request.

## References

[1] Herrman H, Kieling C, McGorry P, Horton R, Sargent J, Patel V (2019) Reducing the global burden of depression: a lancet–world psychiatric association commission. Lancet 393(10189):e42–e43. 10.1016/S0140-6736(18)32408-5 30482607 10.1016/S0140-6736(18)32408-5

[2] Global Burden of Disease 2019 Mental Disorders Collaborators (2022) Global, regional, and national burden of 12 mental disorders in 204 countries and territories, 1990–2019: a systematic analysis for the Global Burden of Disease Study 2019. Lancet Psychiatry 9(2):137–150. 10.1016/S2215-0366(21)00395-3 35026139 10.1016/S2215-0366(21)00395-3 PMC8776563

[3] Arias D, Saxena S, Verguet S (2022) Quantifying the global burden of mental disorders and their economic value. EClinicalMedicine 54:101675. 10.1016/j.eclinm.2022.101675 36193171 10.1016/j.eclinm.2022.101675 PMC9526145

[4] Jacka FN (2017) Nutritional psychiatry: where to next? EBioMedicine. 17:24–29. 10.1016/j.ebiom.2017.02.020 10.1016/j.ebiom.2017.02.020 PMC5360575 28242200

[5] Millar SR, Navarro P, Harrington JM, Shivappa N, Hébert JR, Perry IJ, Phillips CM (2021) Comparing dietary score associations with lipoprotein particle subclass profiles: a cross-sectional analysis of a middle-to older-aged population. Clin Nutr 40(7):4720–4729. 10.1016/j.clnu.2021.06.005 34237699 10.1016/j.clnu.2021.06.005

[6] Lassale C, Batty GD, Baghdadli A, Jacka F, Sanchez-Villegas A, Kivimaki M, Akbaraly T (2019) Healthy dietary indices and risk of depressive outcomes: a systematic review and meta-analysis of observational studies. Mol Psychiatry 24(7):965–986. 10.1038/s41380-018-0237-8 30254236 10.1038/s41380-018-0237-8 PMC6755986

[7] Phillips CM, Shivappa N, Hebert JR, Perry IJ (2018) Dietary inflammatory index and mental health: a cross-sectional analysis of the relationship with depressive symptoms, anxiety and well-being in adults. Clin Nutr 37(5):1485–1491. 10.1016/j.clnu.2017.08.029 28912008 10.1016/j.clnu.2017.08.029

[8] Millar SR, Harrington JM, Perry IJ, Phillips CM (2024) Lifestyle factors and BMI attenuate relationships between biomarkers of inflammation and depressive symptoms and well-being: a cross-sectional study. Brain Behav Immun Health 37:100759. 10.1016/j.bbih.2024.100759 38560580 10.1016/j.bbih.2024.100759 PMC10979065

[9] Willett W, Rockstrom J, Loken B, Springmann M, Lang T, Vermeulen S, Garnett T, Tilman D, DeClerck F, Wood A, Jonell M, Clark M, Gordon LJ, Fanzo J, Hawkes C, Zurayk R, Rivera JA, De Vries W, Majele Sibanda L, Afshin A, Chaudhary A, Herrero M, Agustina R, Branca F, Lartey A, Fan S, Crona B, Fox E, Bignet V, Troell M, Lindahl T, Singh S, Cornell SE, Srinath Reddy K, Narain S, Nishtar S, Murray CJL (2019) Food in the Anthropocene: the EAT-Lancet Commission on healthy diets from sustainable food systems. Lancet 393(10170):447–492. 10.1016/S0140-6736(18)31788-4 30660336 10.1016/S0140-6736(18)31788-4

[10] Elliott PS, Kharaty SS, Phillips CM (2022) Plant-based diets and lipid, lipoprotein, and inflammatory biomarkers of cardiovascular disease: a review of observational and interventional studies. Nutrients 14(24). 10.3390/nu14245371 10.3390/nu14245371 PMC9787709 36558530

[11] Satija A, Bhupathiraju SN, Rimm EB, Spiegelman D, Chiuve SE, Borgi L, Willett WC, Manson JE, Sun Q, Hu FB (2016) Plant-based dietary patterns and incidence of type 2 diabetes in US men and women: results from three prospective cohort studies. PLoS Med 13(6):e1002039. 10.1371/journal.pmed.1002039 27299701 10.1371/journal.pmed.1002039 PMC4907448

[12] Daneshzad E, Keshavarz SA, Qorbani M, Larijani B, Bellissimo N, Azadbakht L (2020) Association of dietary acid load and plant-based diet index with sleep, stress, anxiety and depression in diabetic women. Br J Nutr 123(8):901–912. 10.1017/S0007114519003179 31806069 10.1017/S0007114519003179

[13] Haghighatdoost F, Mahdavi A, Mohammadifard N, Hassannejad R, Najafi F, Farshidi H, Lotfizadeh M, Kazemi T, Karimi S, Roohafza H, Silveira EA, Sarrafzadegan N, de Oliveira C (2023) The relationship between a plant-based diet and mental health: evidence from a cross-sectional multicentric community trial (LIPOKAP study). PLoS ONE 18(5):e0284446. 10.1371/journal.pone.0284446 37256886 10.1371/journal.pone.0284446 PMC10231825

[14] Mousavi SM, Ebrahimi-Mousavi S, Hassanzadeh Keshteli A, Afshar H, Esmaillzadeh A, Adibi P (2022) The association of plant-based dietary patterns and psychological disorders among Iranian adults. J Affect Disord 300:314–321. 10.1016/j.jad.2022.01.028 34990626 10.1016/j.jad.2022.01.028

[15] Zamani B, Daneshzad E, Siassi F, Guilani B, Bellissimo N, Azadbakht L (2020) Association of plant-based dietary patterns with psychological profile and obesity in Iranian women. Clin Nutr 39(6):1799–1808. 10.1016/j.clnu.2019.07.019 31399262 10.1016/j.clnu.2019.07.019

[16] Kearney PM, Harrington JM, Mc Carthy VJ, Fitzgerald AP, Perry IJ (2013) Cohort profile: the cork and kerry diabetes and heart disease study. Int J Epidemiol 42(5):1253–1262. 10.1093/ije/dys131 22984148 10.1093/ije/dys131

[17] Riboli E, Elmståhl S, Saracci R, Gullberg B, Lindgärde F (1997) The malmö food study: validity of two dietary assessment methods for measuring nutrient intake. Int J Epidemiol 26(suppl1):S161. 10.1093/ije/26.suppl_1.s161 9126544 10.1093/ije/26.suppl_1.s161

[18] Harrington JM (1997) Validation of a food frequency questionnaire as a tool for assessing nutrient intake. NUI, Galway

[19] Elliott PS, Harrington JM, Millar SR, Otvos JD, Perry IJ, Phillips CM (2023) Plant-based diet indices and lipoprotein particle subclass profiles: a cross-sectional analysis of middle- to older-aged adults. Atherosclerosis 380:117190. 10.1016/j.atherosclerosis.2023.117190 37552902 10.1016/j.atherosclerosis.2023.117190

[20] Lenore Sawyer Radloff (1977) The CES-D scale: a self-report depression scale for research in the general population. Appl Psychol Meas 1(3):385–401. 10.1177/014662167700100306

[21] Zigmond AS, Snaith RP (1983) The hospital anxiety and depression scale. Acta psychiatrica Scandinavica 67(6):361–370. 10.1111/j.1600-0447.1983.tb09716.x 6880820 10.1111/j.1600-0447.1983.tb09716.x

[22] World Health Organization (1998) Wellbeing measures in health care: the depcare project: report on a WHO meeting Stockholm, København, Sweden, 12–13 February 1998. World Health Organization. Regional Office for Europe

[23] Craig CL, Marshall AL, Sjöström M, Bauman AE, Booth ML, Ainsworth BE, Pratt M, Ekelund U, Yngve A, Sallis JF (2003) International physical activity questionnaire: 12-country reliability and validity. Med Sci Sports Exerc 35(8):1381–1395. 10.1249/01.MSS.0000078924.61453.FB 12900694 10.1249/01.MSS.0000078924.61453.FB

[24] Khaw K-T, Wareham N, Bingham S, Welch A, Luben R, Day N (2008) Combined impact of health behaviours and mortality in men and women: the EPIC-Norfolk prospective population study. PLoS Med 5(1):e12. 10.1371/journal.pmed.0050012 18184033 10.1371/journal.pmed.0050012 PMC2174962

[25] American Diabetes Association (2013) Diagnosis and classification of diabetes mellitus. Diabetes Care 36(Suppl 1):S67–S74. 10.2337/dc13-S067 23264425 10.2337/dc13-S067 PMC3537273

[26] Shafiei F, Salari-Moghaddam A, Larijani B, Esmaillzadeh A (2023) Mediterranean diet and depression: reanalysis of a meta-analysis. Nutr Rev 81(7):889–890. 10.1093/nutrit/nuad023 36928725 10.1093/nutrit/nuad023

[27] Meegan AP, Perry IJ, M. PC (2017) The association between dietary quality and dietary guideline adherence with mental health outcomes in adults: a cross-sectional analysis. Nutrients 9(3):238. 10.3390/nu9030238 28273871 10.3390/nu9030238 PMC5372901

[28] Kharaty S, Harrington JM, Millar SR, Perry IJ, Phillips CM (2023) Plant-based dietary indices and biomarkers of chronic low-grade inflammation: a cross-sectional analysis of adults in Ireland. Eur J Nutr 62(8):3397–3410. 10.1007/s00394-023-03242-5 37658860 10.1007/s00394-023-03242-5 PMC10611858

[29] Quek J, Lim G, Lim WH, Ng CH, So WZ, Toh J, Pan XH, Chin YH, Muthiah MD, Chan SP, Foo RSY, Yip J, Neelakantan N, Chong MFF, Loh PH, Chew NWS (2021) The association of plant-based diet with cardiovascular disease and mortality: a meta-analysis and systematic review of prospect cohort studies. Front Cardiovasc Med 8:756810. 10.3389/fcvm.2021.756810 34805312 10.3389/fcvm.2021.756810 PMC8604150

[30] Lee MF, Eather R, Best T (2021) Plant-based dietary quality and depressive symptoms in Australian vegans and vegetarians: a cross-sectional study. BMJ Nutr Prev Health 4(2):479–486. 10.1136/bmjnph-2021-000332 35028517 10.1136/bmjnph-2021-000332 PMC8718860

[31] Vasmehjani AA, Darabi Z, Ghayour-Mobarhan M, Ferns GA, Khayyatzadeh SS (2024) The associations between plant-based dietary indices with depression and quality of life and insomnia among Iranian adolescent girls in 2015. Sci Rep 14(1):11683. 10.1038/s41598-024-61952-0 38778083 10.1038/s41598-024-61952-0 PMC11111745

[32] Cacau LT, De Carli E, de Carvalho AM, Lotufo PA, Moreno LA, Bensenor IM, Marchioni DM (2021) Development and validation of an index based on EAT-Lancet recommendations: the planetary health diet index. Nutrients 13(5). 10.3390/nu13051698 10.3390/nu13051698 PMC8156093 34067774

[33] Kamrani F, Kachouei AA, Sobhani SR, Khosravi M (2024) Nourishing the mind: how the EAT-Lancet reference diet (ELD) and MIND diet impact stress, anxiety, and depression. BMC Psychiatry 24(1):709. 10.1186/s12888-024-06165-5 39427151 10.1186/s12888-024-06165-5 PMC11490120

[34] Carey MT, Millar SR, Elliott PS, Navarro P, Harrington JM, Perry IJ, Phillips CM (2024) Plant-based diet adherence is associated with metabolic health status in adults living with and without obesity. Eur J Nutr 63(6):2235–2246. 10.1007/s00394-024-03399-7 38753172 10.1007/s00394-024-03399-7 PMC11377579

[35] Lai JS, Hiles S, Bisquera A, Hure AJ, McEvoy M, Attia J (2014) A systematic review and meta-analysis of dietary patterns and depression in community-dwelling adults. Am J Clin Nutr 99(1):181–197. 10.3945/ajcn.113.069880 24196402 10.3945/ajcn.113.069880

[36] Millar SR, Harrington JM, Perry IJ, Phillips CM (2025) Associations between ultra-processed food and drink consumption and biomarkers of chronic low-grade inflammation: exploring the mediating role of adiposity. Eur J Nutr 64(4):150. 10.1007/s00394-025-03666-1 40205185 10.1007/s00394-025-03666-1 PMC11982146

[37] Millar SR, Harrington JM, Perry IJ, Phillips CM (2022) Associations between a protective lifestyle behaviour score and biomarkers of chronic low-grade inflammation: a cross-sectional analysis in middle-to-older aged adults. Int J Obes 46(3):476–485. 10.1038/s41366-021-01012-z 10.1038/s41366-021-01012-z 34744162

[38] Lonergan C, Millar SR, Kabir Z (2024) Associations between adiposity measures and depression and well-being scores: a cross-sectional analysis of middle-to older-aged adults. PLoS ONE 19(3):e0299029. 10.1371/journal.pone.0299029 38446756 10.1371/journal.pone.0299029 PMC10917308

