# Supplementary material for: Plant-based dietary indices and mental health: a cross-sectional study of a middle- to older-aged population
Source: Eur J Nutr. 2026 May 26;65(4):139. doi: 10.1007/s00394-026-04000-z (PMC13212728; doi:10.1007/s00394-026-04000-z)
Supplement: Supplementary file 1 — Supplementary Material 1 [file 394_2026_4000_MOESM1_ESM.docx]

| **Supplementary Table 1** Food items from the European Prospective Investigation into Cancer and Nutrition 150-item semi-quantitative food frequency questionnaire (modified for the Irish population) constituting the 18 food groups used in the plant-based diet indices | | | | |
| --- | --- | --- | --- | --- |
| **Plant Food Groups** | **Food Items** | **oPDI** | **hPDI** | **uPDI** |
| **Healthy** |  |  |  |  |
| Whole grains | Brown bread, wholemeal bread, porridge, All-Bran (cereal), Bran Flakes (cereal), muesli, brown rice, wholemeal pasta | Positive scores | Positive scores | Reverse scores |
| Fruits | Apples, pears, oranges, grapefruit, bananas, grapes, melon, peaches, strawberries, tinned fruit, dried fruit, frozen fruit | Positive scores | Positive scores | Reverse scores |
| Vegetables | Carrots, spinach, broccoli, brussels sprouts, cabbage, green beans, courgette (zucchini), cauliflower, parsnips, leeks, onions, garlic, mushrooms, sweet peppers, beansprouts, green salad vegetables, cucumber, watercress, tomatoes, sweetcorn, beetroot, avocado, tomato sauce, pickles | Positive scores | Positive scores | Reverse scores |
| Nuts | Nuts, peanut butter | Positive scores | Positive scores | Reverse scores |
| Legumes | Peas, beans, lentils, soya meat (including tofu, veggie burger), soya milk | Positive scores | Positive scores | Reverse scores |
| Vegetable oils | French dressing, low-fat margarine, cholesterol-lowering margarine, vegetable oil-based spreads, olive oil spreads, vegetable oil/cream-based spreads | Positive scores | Positive scores | Reverse scores |
| Tea/coffee | Tea, coffee, decaffeinated coffee | Positive scores | Positive scores | Reverse scores |
| **Unhealthy** |  |  |  |  |
| Fruit juices | Pure fruit juices (e.g., orange juice, grapefruit juice, other fruit juice) | Positive scores | Reverse scores | Positive scores |
| Refined grains | White bread, cream crackers, crisp bread, pancakes, cornflakes, sugar-coated cereals, white rice, white pasta, home-baked buns (e.g., scones, flapjacks) | Positive scores | Reverse scores | Positive scores |
| Potatoes | Boiled or baked potatoes, mashed potatoes, chips (French fries), roast potatoes, potato salad, crisps (potato chips) | Positive scores | Reverse scores | Positive scores |
| Sugar-sweetened beverages | Carbonated soft drinks (e.g., Coca-Cola, lemonade), fruit squash or cordial | Positive scores | Reverse scores | Positive scores |
| Sweets/desserts | Chocolate biscuits (cookies), plain biscuits (cookies), cakes, ready-made buns (e.g., croissants, doughnuts), chocolate, sweets (confectionaries), sugar, jam, marmalade, honey, syrup | Positive scores | Reverse scores | Positive scores |
| **Animal Food Groups** |  |  |  |  |
| Animal fat | Butter, light butter | Reverse scores | Reverse scores | Reverse scores |
| Dairy | Whole milk, low-fat milk, skimmed milk, high-low milk, buttermilk, dried milk, cream, full-fat yoghurt, low-fat yoghurt, dairy-based desserts, cheese, milk puddings, ice cream, cocoa or Horlick’s (made with milk) | Reverse scores | Reverse scores | Reverse scores |
| Egg | Eggs | Reverse scores | Reverse scores | Reverse scores |
| Fish/seafood | White fish (fresh or frozen), oily fish (fresh or canned), fried fish in batter, fish fingers, shellfish | Reverse scores | Reverse scores | Reverse scores |
| Poultry/red meat | Beef (including hamburgers, corned beef), mixed dishes with beef, mixed dishes with pork (including bacon, ham, sausages), chicken or turkey (including breaded chicken), lamb, mixed dishes with lamb, liver (including pâté), heart, kidney | Reverse scores | Reverse scores | Reverse scores |
| Miscellaneous animal-based foods | Savoury meat pie, meat or vegetable lasagne (containing cheese/milk), moussaka, pizza, macaroni and cheese, quiche, mayonnaise, coleslaw (containing mayonnaise and cream), meat soup (homemade/tinned) | Reverse scores | Reverse scores | Reverse scores |
| **Abbreviations:** oPDI: Overall Plant-Based Diet Index; hPDI: Healthy Plant-Based Diet Index; uPDI: Unhealthy Plant-Based Diet Index.  The following foods were excluded from analysis as they did not fit into any of the 18 food group categories: other dressings, Canderel (artificial sweetener), curry sauce, other sauces, Marmite (paste made from yeast extract), Coffee Mate (coffee whitener). Tinned vegetable soup was excluded because these products may contain dairy and excess sodium/sugar which makes it dissimilar to other foods included in the ‘Vegetables’ category. Regardless, intakes of the aforementioned food groups were minimal (median for other sauces = 0.14 servings/d; median for all other food groups = 0 servings/d). Adapted from Elliott et al., [19]. | | | | |

**Supplementary Table 2** Linear regression analysis of oPDI, hPDI and uPDI quartile associations with mental health scores

|  | **oPDI Q1** | **oPDI Q2** | **oPDI Q3** | **oPDI Q4** |  |
| --- | --- | --- | --- | --- | --- |
| **Depression (CES-D score)** |  | **β (95% CI)** | **β (95% CI)** | **β (95% CI)** | ***p* trend** |
| Model 1 | [reference] | -1.570 (-2.529, -0.611) | -1.278 (-2.267, -0.290) | -1.297 (-2.294, -0.300) | **0.025** |
| Model 2 | [reference] | -1.479 (-2.413, -0.544) | -1.290 (-2.261, -0.319) | -1.488 (-2.500, -0.475) | **0.009** |
| Model 3 | [reference] | -1.405 (-2.335, -0.475) | -1.204 (-2.172, -0.236) | -1.373 (-2.380, -0.365) | **0.016** |
| Model 4 | [reference] | -1.402 (-2.328, -0.475) | -1.118 (-2.085, -0.152) | -1.306 (-2.311, -0.301) | **0.025** |
| **Anxiety (HADS-A score)** |  |  |  |  |  |
| Model 1 | [reference] | -0.216 (-0.637, 0.205) | -0.336 (-0.770, 0.098) | -0.392 (-0.829, 0.045) | 0.066 |
| Model 2 | [reference] | -0.211 (-0.626, 0.205) | -0.361 (-0.793, 0.072) | -0.473 (-0.923, -0.239) | **0.031** |
| Model 3 | [reference] | -0.179 (-0.595, 0.237) | -0.320 (-0.754, 0.113) | -0.438 (-0.888, 0.012) | **0.045** |
| Model 4 | [reference] | -0.180 (-0.595, 0.236) | -0.286 (-0.719, 0.148) | -0.416 (-0.865, 0.034) | 0.063 |
| **Well-being (WHO-5 score)** |  |  |  |  |  |
| Model 1 | [reference] | 0.115 (-0.526, 0.756) | 0.566 (-0.091, 1.223) | 0.623 (-0.038, 1.284) | **0.03** |
| Model 2 | [reference] | 0.051 (-0.585, 0.688) | 0.525 (-0.134, 1.185) | 0.635 (-0.051, 1.321) | **0.032** |
| Model 3 | [reference] | 0.028 (-0.605, 0.661) | 0.479 (-0.177, 1.136) | 0.586 (-0.097, 1.269) | **0.046** |
| Model 4 | [reference] | 0.025 (-0.608, 0.658) | 0.433 (-0.225, 1.091) | 0.551 (-0.132, 1.234) | 0.063 |
|  | **hPDI Q1** | **hPDI Q2** | **hPDI Q3** | **hPDI Q4** |  |
| **Depression (CES-D score)** |  | **β (95% CI)** | **β (95% CI)** | **β (95% CI)** | ***p* trend** |
| Model 1 | [reference] | -0.220 (-1.176, 0.735) | -1.285 (-2.252, -0.318) | -1.614 (-2.621, -0.607) | **<0.001** |
| Model 2 | [reference] | -0.450 (-1.393, 0.493) | -1.302 (-2.276, -0.328) | -1.429 (-2.474, -0.384) | **0.003** |
| Model 3 | [reference] | -0.269 (-1.208, -0.670) | -1.040 (-2.014, -0.067) | -1.076 (-2.122, -0.030) | **0.019** |
| Model 4 | [reference] | -0.260 (-1.120, 0.076) | -1.025 (-1.995, -0.055) | -1.100 (-2.143, -0.057) | **0.017** |
| **Anxiety (HADS-A score)** |  |  |  |  |  |
| Model 1 | [reference] | -0.008 (-0.427, 0.411) | -0.202 (-0.627, 0.223) | -0.289 (-0.731, 0.153) | 0.138 |
| Model 2 | [reference] | -0.100 (-0.519, 0.318) | -0.234 (-0.667, 0.200) | -0.273 (-0.738, 0.192) | 0.207 |
| Model 3 | [reference] | -0.058 (-0.477, 0.361) | -0.163 (-0.598, 0.272) | -0.176 (-0.644, 0.291) | 0.404 |
| Model 4 | [reference] | -0.037 (-0.456, 0.382) | -0.155 (-0.590, 0.280) | -0.176 (-0.644, 0.291) | 0.393 |
| **Well-being (WHO-5 score)** |  |  |  |  |  |
| Model 1 | [reference] | 0.297 (-0.341, 0.936) | 0.411 (-0.234, 1.056) | 0.632 (-0.042, 1.305) | 0.065 |
| Model 2 | [reference] | 0.459 (-0.183, 1.100) | 0.481 (-0.181, 1.144) | 0.629 (-0.081, 1.338) | 0.098 |
| Model 3 | [reference] | 0.350 (-0.288, 0.989) | 0.324 (-0.338, 0.986) | 0.419 (-0.291, 1.130) | 0.286 |
| Model 4 | [reference] | 0.331 (-0.308, 0.970) | 0.313 (-0.348, 0.975) | 0.426 (-0.285, 1.137) | 0.274 |
|  | **uPDI Q1** | **uPDI Q2** | **uPDI Q3** | **uPDI Q4** |  |
| **Depression (CES-D score)** |  | **β (95% CI)** | **β (95% CI)** | **β (95% CI)** | ***p* trend** |
| Model 1 | [reference] | 0.482 (-0.464, 1.428) | 0.458 (-0.550, 1.465) | 0.791 (-0.223, 1.805) | 0.152 |
| Model 2 | [reference] | 0.298 (-0.632, 1.228) | 0.401 (-0.608, 1.409) | 0.936 (-0.119, 1.990) | 0.089 |
| Model 3 | [reference] | 0.254 (-0.669, 1.177) | 0.121 (-0.884, 1.127) | 0.533 (-0.523, 1.590) | 0.392 |
| Model 4 | [reference] | 0.287 (-0.634, 1.207) | 0.238 (-0.768, 1.244) | 0.577 (-0.477, 1.630) | 0.326 |
| **Anxiety (HADS-A score)** |  |  |  |  |  |
| Model 1 | [reference] | 0.082 (-0.332, 0.496) | -0.007 (-0.448, 0.433) | -0.079 (-0.524, 0.366) | 0.65 |
| Model 2 | [reference] | -0.009 (-0.422, 0.404) | -0.094 (-0.541, 0.354) | -0.144 (-0.613, 0.325) | 0.497 |
| Model 3 | [reference] | -0.021 (-0.433, 0.391) | -0.169 (-0.617, 0.279) | -0.261 (-0.734, 0.211) | 0.244 |
| Model 4 | [reference] | -0.011 (-0.423, 0.401) | -0.119 (-0.568, 0.331) | -0.240 (-0.712, 0.232) | 0.28 |
| **Well-being (WHO-5 score)** |  |  |  |  |  |
| Model 1 | [reference] | -0.366 (-1.000, 0.267) | -0.134 (-0.806, 0.538) | -0.272 (-0.947, 0.403) | 0.593 |
| Model 2 | [reference] | -0.239 (-0.873, 0.396) | -0.039 (-0.725, 0.648) | -0.183 (-0.901, 0.535) | 0.765 |
| Model 3 | [reference] | -0.214 (-0.844, 0.416) | 0.132 (-0.553, 0.816) | 0.075 (-0.645, 0.795) | 0.626 |
| Model 4 | [reference] | -0.229 (-0.858, 0.401) | 0.075 (-0.611, 0.762) | 0.055 (-0.665, 0.775) | 0.689 |

Abbreviations: CES-D: Center for Epidemiologic Studies Depression; HADS-A: Hospital Anxiety and Depression Scale-Anxiety; WHO-5: World Health Organization-Five Well-Being Index.

oPDI: Overall Plant-Based Diet Index; hPDI: Healthy Plant-Based Diet Index; uPDI: Unhealthy Plant-Based Diet Index.

Model 1: adjusted for age and sex.

Model 2: adjusted for age, sex, energy intake, BMI and anti-depressant use.

Model 3: adjusted for age, sex, energy intake, BMI, anti-depressant use, education, smoking, alcohol use and physical activity.

Model 4: additionally adjusted for history of type 2 diabetes, cardiovascular disease and cancer.

Beta coefficients (β) and 95% confidence intervals (CI) are shown. Significant *p* shown in **bold**.

**Supplementary Table 3** Logistic regression analysis of oPDI and hPDI quartile associations with clinical depression (CES-D score cut-off >16)

|  | **PDI** |  | **hPDI** |  |
| --- | --- | --- | --- | --- |
|  | **OR (95% CI)** | ***p* trend** | **OR (95% CI)** | ***p* trend** |
| **Model 1** |  |  |  |  |
| Q4 | [reference] | 0.23 | [reference] | **0.022** |
| Q3 | 0.842 (0.580, 1.222) |  | 0.942 (0.634, 1.399) |  |
| Q2 | 0.872 (0.608, 1.251) |  | 1.555 (1.075, 2.250) |  |
| Q1 | 1.236 (0.877, 1.742) |  | 1.330 (0.921, 1.920) |  |
| **Model 2** |  |  |  |  |
| Q4 | [reference] | 0.086 | [reference] | 0.112 |
| Q3 | 0.902 (0.612, 1.331) |  | 0.856 (0.569, 1.286) |  |
| Q2 | 0.969 (0.662, 1.418) |  | 1.353 (0.919, 1.992) |  |
| Q1 | 1.384 (0.952, 2.014) |  | 1.213 (0.811, 1.815) |  |
| **Model 3** |  |  |  |  |
| Q4 | [reference] | 0.103 | [reference] | 0.257 |
| Q3 | 0.893 (0.604, 1.321) |  | 0.831 (0.551, 1.252) |  |
| Q2 | 0.954 (0.650, 1.399) |  | 1.306 (0.884, 1.930) |  |
| Q1 | 1.367 (0.936, 1.996) |  | 1.105 (0.734, 1.663) |  |
| **Model 4** |  |  |  |  |
| Q4 | [reference] | 0.132 | [reference] | 0.235 |
| Q3 | 0.892 (0.602, 1.322) |  | 0.844 (0.559, 1.274) |  |
| Q2 | 0.935 (0.637, 1.374) |  | 1.325 (0.895, 1.961) |  |
| Q1 | 1.344 (0.918, 1.967) |  | 1.120 (0.743, 1.690) |  |

Abbreviations: CES-D: Center for Epidemiologic Studies Depression; oPDI: Overall Plant-Based Diet Index;

hPDI: Healthy Plant-Based Diet Index.

Model 1: adjusted for age and sex.

Model 2: adjusted for age, sex, energy intake, BMI and anti-depressant use.

Model 3: adjusted for age, sex, energy intake, BMI, anti-depressant use, education, smoking, alcohol use and physical activity.

Model 4: additionally adjusted for history of type 2 diabetes, cardiovascular disease and cancer.

Odds ratios (OR) and 95% confidence intervals (CI) are shown. Significant *p* shown in **bold**.
